# Comparative Analysis of the Safety and Functional Outcomes of Anterior versus Retropupillary Iris-Claw IOL Fixation

**DOI:** 10.1155/2018/8463569

**Published:** 2018-11-04

**Authors:** Paolo Mora, Giacomo Calzetti, Stefania Favilla, Matteo Forlini, Salvatore Tedesco, Purva Date, Viola Tagliavini, Arturo Carta, Rino Frisina, Emilio Pedrotti, Stefano Gandolfi

**Affiliations:** ^1^Ophthalmology Unit, University Hospital of Parma, Parma, Italy; ^2^Independent Researcher, Parma, Italy; ^3^Aditya Jyot Eye Hospital, Wadala, Mumbai, India; ^4^Department of Ophthalmology, University of Padova, Padova, Italy; ^5^Eye Clinic, Department of Neurosciences, Biomedicine and Movement Sciences, University of Verona, AOUI-Policlinico G. B. Rossi, Verona, Italy

## Abstract

**Purpose:**

To compare the functional and clinical outcomes of the iris-claw intraocular lens (IOL) placed on the anterior versus posterior surface of the iris.

**Patients and Methods:**

A multicenter, retrospective study. Data on eyes that underwent anterior or retropupillary iris-claw IOL implantation because of inadequate capsular support secondary to complicated cataract surgery, trauma, and dislocated/opacified IOLs since January 2015 were analyzed. For study inclusion, evaluation results had to be available in the medical records both preoperatively and at 1 and 12 months after implantation. The following parameters were compared between the groups: best-corrected distance visual acuity (BCDVA), spherical and cylindrical refractive error, endothelial cell density (ECD), central macular thickness (CMT), and percentage and type of postoperative complications.

**Results:**

In total, 60 eyes of 60 patients aged 73 ± 13 years were included: 28 eyes (47%) involved anterior, and 32 eyes (53%) retropupillary, iris-claw IOL fixations. Preoperatively, the groups were similar in all parameters except for a significantly higher proportion of retropupillary fixations in patients who had previously experienced a closed-globe trauma (*p*=0.03). The groups showed comparable improvements in BCDVA after surgery (final BCDVA: 0.34 ± 0.45 vs. 0.37 ± 0.50 logMAR in the anterior and retropupillary placement groups, respectively). During follow-up, no group difference was observed in refractive error or CMT. Both groups experienced similarly marked ECD loss and showed similar incidence of postoperative complications, with cystoid macular edema being the most common complication. Multivariable linear regression showed that BCDVA at 1 month was the best predictor of the final BCDVA.

**Conclusions:**

Anterior chamber and posterior chamber iris-claw IOL fixations proved equally effective and safe for aphakic correction in eyes with inadequate capsular support.

## 1. Introduction

Management of intraocular lens (IOL) implantation in eyes with inadequate capsular support is challenging. Inadequate capsular support precluding the availability of the natural bag or of the sulcus can result from complicated cataract surgery, luxation of the crystalline lens, or from dislocation or opacification of a previous conventional IOL. Various methods of surgical correction have been described, including placement of specialized IOLs supported by the anterior chamber (AC) angle or iris or of scleral-fixated IOLs [1–3]. Iris-fixated IOLs secured to the anterior surface of the iris using a claw-shaped haptic device were initially used for correction of aphakia [4]. Although this type of IOL was used extensively in the past, it is no longer recommended because of relatively high complication rates and suboptimal visual outcomes [5, 6]. However, anterior iris-claw IOLs have since undergone significant design changes, including vault modifications, which have enabled their use for refractive correction of high myopia in phakic eyes. These advances, along with the ease of surgical insertion, have led to the reintroduction of anterior iris-claw IOLs for correction of aphakia without a capsular support [7, 8]. Iris-claw IOLs can also be securely fixed to the posterior surface of the iris, to maintain the physiological position of the diaphragm in the posterior chamber (PC) of the eye and thus reduce the potential for the complications associated with AC IOLs. Many investigators have reported relatively large series, both prospective and retrospective, demonstrating the midterm safety and efficacy of this procedure [9–12]. Both AC and PC iris-claw IOLs were also found to be safe and effective for visual rehabilitation in young patients [13, 14]. Iris-claw fixation, both anterior and retropupillary, is technically less demanding than scleral fixation and is now used in routine practice by many surgeons. Only one study has prospectively compared the visual outcomes between the anterior and retropupillary approaches for fixation of iris-claw IOLs, wherein no difference was noted [15]. However, the study was limited by a small sample size and short follow-up. The present study aimed to supplement the available data by retrospectively investigating long-term safety and visual outcomes in a larger cohort of eyes implanted with either AC or PC iris-claw IOLs.

## 2. Methods

**Figure 1:**
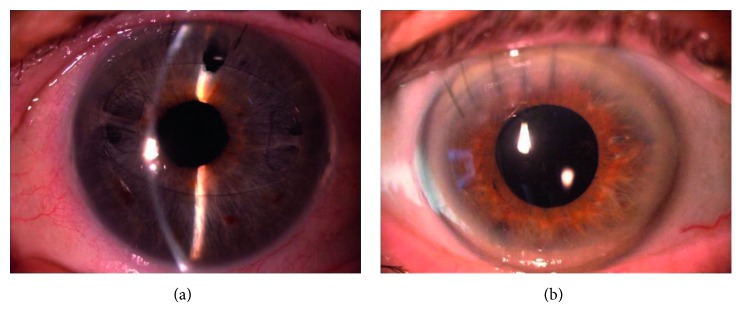
Slit-lamp photographs of iris-claw IOLs implanted in the anterior chamber (a) and in the posterior chamber (b).

This was a three-center retrospective study of patients who received AC or PC iris-claw IOL implants from January 2015. Informed consent was obtained from all patients, and all procedures adhered to the tenets of the Declaration of Helsinki. The inclusion criteria were (a) uneventful implantation (i.e., absence of intraoperative complications such as iridodialysis, iris bleeding, or intraoperative disenclavation) of an Artisan® (Ophtec, Groningen, The Netherlands) iris-claw IOL, inserted due to a lack of capsule support; and (b) medical records showing the results of complete eye examinations (as detailed below), preoperatively and at 1 and 12 months after surgery. Windows of ±1 and ±4 weeks were allowed for the evaluations at 1 and 12 months, respectively. The exclusion criterion was any severe media opacity precluding examination of the ocular structures. Complete examination results had to be available within the medical records, including an up-to-date medical history; refraction assessment; best-corrected distance visual acuity (BCDVA; assessed by the standard Early Treatment of Diabetic Retinopathy Study chart); slit lamp examination, fundus evaluation, and intraocular pressure (IOP) results; corneal endothelial cell density (ECD) count; and macular optical coherence tomography (OCT) data. Included eyes (one per patient) were divided into two groups according to the implant position: anterior (group A) or posterior (group B) (Figure 1). The following parameters were compared between the groups: BCDVA (given by the logarithm of the minimum angle of resolution, logMAR), the spherical and cylindrical refractive error (considered separately after conversion to positive cylinders), ECD, central macular thickness (CMT), and the percentage and type of postoperative complications and/or anomalies. These data were collected by an experienced clinician; either an ophthalmologist or a certified ophthalmic technician. ECD was expressed as the number of cells per mm^2^ and was measured in all patients with a specular microscope (SP-2000P; Topcon America, Paramus, NJ, USA) using the “center-dot” cell counting method. CMT was assessed in all patients, according to the central subfield thickness, via “macular cube 512 × 128” OCT scans performed with the Cirrus HD-OCT 4000 instrument (Carl Zeiss Meditec, Dublin, CA, USA).

### 2.1. Surgical Techniques

An Artisan® (Ophtec) IOL was used during the surgery; Artisan® is a rigid poly(methyl methacrylate) IOL 8.5 mm in length and with a maximum height of 1.04 mm and an optical zone width of 5.4 mm. The IOL power was calculated using the SRK/T formula. The IOL targeted emmetropia in all patients. An A-constant of 115.0, as per the manufacturer's recommendation, was used for anterior implantation, while an A-constant of 116.5 was used for retropupillary fixation. Anesthesia was either general or peribulbar. Whenever indicated, the surgical procedure was combined with lens/IOL removal or pars plana vitrectomy (PPV). Surgeons selected either AC or PC placement depending on their individual experience and the characteristics of the specific surgical procedure, in particular the presence/necessity of a vitreous access which could allow to handle the implant even from behind by a vitreous pick as a rescue measure in the case of unsuitable retropupillary iris hooking. The same standardized surgical technique was applied in both procedures. Two side ports were made at the 3 and 9 o'clock positions. Anterior vitrectomy was performed as required. Miosis was achieved using intracameral carbachol, and a viscoelastic agent was injected into the anterior chamber. A 5.5 mm superior limbal corneal incision was made, and the IOL (with the vault facing up and down for the anterior clawed lens and retropupillary lens, respectively) was inserted into the AC. The IOL was rotated such that the haptics were lined at the 3 and 9 o'clock positions. Thereafter, the IOL optic plate was held in place with Artisan lens forceps (Ophtec); for AC implantation, iris was enclavated at midperiphery between claw haptics using a special enclavation microspatula introduced through the ipsilateral side port. For retropupillary fixation, after positioning one haptic of the IOL behind the iris, it was enclavated using the microspatula, followed by enclavation of the other haptic. Superior peripheral iridectomy was performed only for AC implantations. Finally, the corneal incision was sutured using noncontinuous 10–0 nonabsorbable nylon sutures, which were removed at a minimum of 6 weeks after surgery. Postoperative therapy included antibiotic and nonsteroidal anti-inflammatory eye drops for 1 month.

### 2.2. Statistical Analysis

Continuous variables were expressed as means ± standard deviation (SD) or as medians with the interquartile range (IQR), while categorical variables were presented as proportions. Differences between groups in the preoperative characteristics (Table 1) and in postoperative complications (Table 2) were analyzed using Student's *t*-test for continuous variables and the chi-squared test or Fisher's exact test for categorical variables. The study outcomes BCDVA, CMT, and ECD loss were analyzed with a two-way ANOVA model (main effect: Group and Time) with interaction term (Group *∗* Time). The program R cran, ver. 3.4.0 was used to perform the analyses. For post hoc analysis, we used a Fisher's Least Significant Difference (LSD) test (95% family-wise confidence level). Univariate and multivariable linear regression analyses were performed to evaluate factors influencing the final BCDVA. Covariates significant at *p* < 0.1 in univariate analyses were included in the multivariable analyses, as were those showing variance inflation ≤10 or having clinical relevance for the study (e.g., anterior vs. posterior implantation). Best-fit regression models were created based on stepwise forward and backward methods. Correlations between continuous variables are described by LOWESS curves and Pearson's correlation coefficients. All data were entered into Excel software (Microsoft Corp., Redmond, WA, USA) and analyzed using Stata software (ver. 12.0; Stata Corp., Fort Worth, TX, USA). A *p* value of <0.05 was considered statistically significant.

## 3. Results

**Figure 2:**
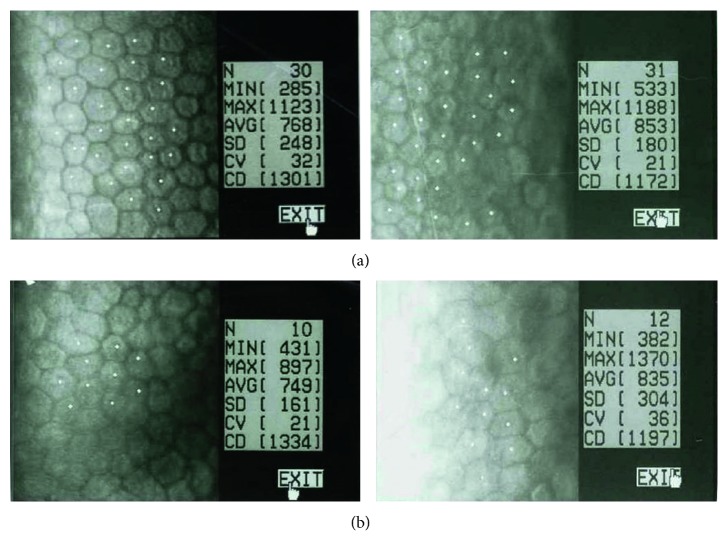
Corneal specular microscopy pictures for both groups. (a) On the left, preoperative endothelial cell density (ECD) and on the right, ECD of the same patient in group A 1 month after surgery. (b) Same sequence in a patient in group B.

**Figure 3:**
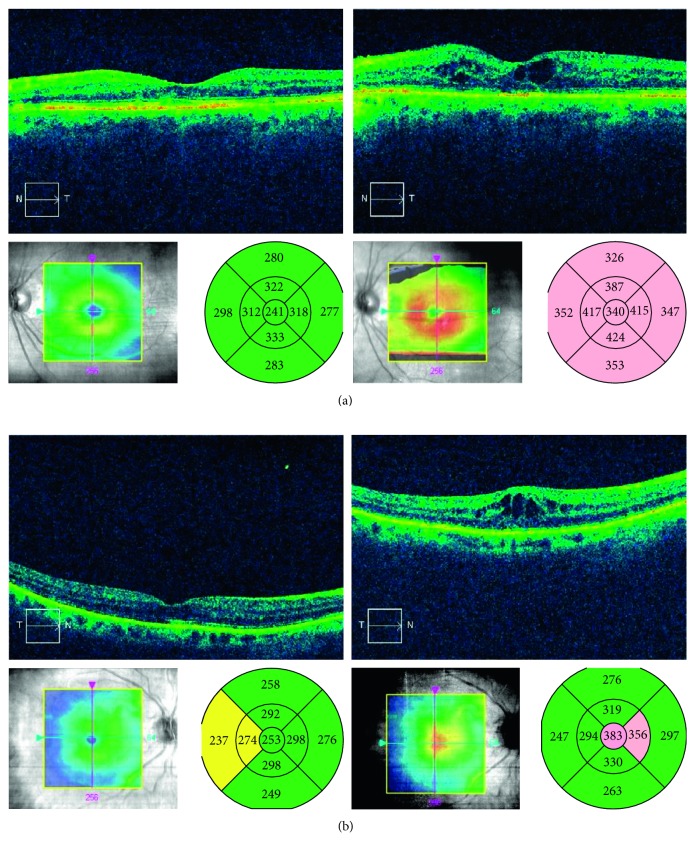
Optical coherence tomography (OCT) scans of cystoid macular edema (CME). (a) OCT scans of a patient in group A at the preoperative visit (left) and at the 1-year postoperative visit, when CME was detected. (b) OCT scans of a patient in group B showing normal foveal thickness at the preoperative visit and CME at the 1-month postoperative visit.

Sixty eyes of sixty subjects (41 males and 19 females; mean age, 73 ± 13 years) were included in the study. In total, 28 eyes (47%) with anterior iris-claw IOL fixation were assigned to group A, and 32 eyes (53%) with retropupillary fixation were assigned to group B. The demographic and preoperative characteristics of the two groups are listed in Table 1. The groups were similar on all parameters, except for a significantly higher proportion of retropupillary fixations in patients who had previously experienced a closed-globe trauma (*p*=0.03). Iris-claw IOL fixation directly followed lens removal in 25 eyes (42% of cases): the indications were complicated cataract surgery in 15 patients and lens dislocation because of trauma or pseudoexfoliative syndrome in 10 patients; the remaining cases were secondary implantations in aphakic eyes (18 cases, 30%) or conventional IOL exchanges because of subluxation/dislocation or opacification (17 cases, 28%). Overall, PPV was performed in combination with iris-claw IOL fixation in 38 eyes (no difference in the rate of performance between the two groups). According to the within-group analysis shown in Table 3, BCDVA significantly improved after surgery in both groups, without significant difference between the two groups. The post hoc analysis showed that the improvement was statistically significant in the time intervals: preop.: 1 month, and preop.: 12 months. At 12 months, spherical and cylindrical refractive errors were also comparable between the two groups. Preoperative OCT data were available, and of suitable quality, in 48 patients (21 in group A and 27 in group B): CMT data and the incidence of CME referred to this subgroup of patients accordingly; the CMT did not significantly change after surgery in either group (Table 3). To determine the ECDs, ten eyes (seven in group B) were excluded from the analysis because of a prior corneal surgery (corneal transplantation or corneal wound suture) or because they developed corneal decompensation during the follow-up. Compared with the preoperative assessments, the ECDs were significantly reduced in both groups after surgery, without a significant intergroup difference (Table 3); at the 1-month postoperative visit, the median losses were 194 cells/mm^2^ (IQR: 65–554 cells/mm^2^) and 203 cells/mm^2^ (IQR: 93–755 cells/mm^2^) in groups A and B, respectively, with no intergroup difference (*p*=0.51). The ECD showed a further decrease at the final evaluation; the median losses between 1 and 12 months were 90 cells/mm^2^ (IQR: 7–221 cells/mm^2^) and 109 cells/mm^2^ (IQR: 15–209 cells/mm^2^) in groups A and B, respectively, with no intergroup difference (*p*=0.89) (Figure 2). Again, the post hoc analysis showed that the loss was statistically significant in the time intervals: preop.: 1 month, and preop.: 12 months. The most frequent complications in both groups throughout the postoperative follow-up were cystoid macular edema (CME) (Figure 3) and transiently increased IOP. CME was detected in 5 subjects at the 1-month visit (1 patient in group A and 4 in group B). At the 1-year visit, CME was detected in 10 subjects (6 in group A and 4 in group B), with one patient in group B showing CME at both the postoperative visits. The cumulative incidence of CME over 12 months after surgery was not significantly different between the two groups. CME occurred in 6 patients who underwent PPV and in 8 without PPV. The treatment of CME included nonsteroidal anti-inflammatory eye drops in seven cases, prednisolone eye drops in one case, and subtenon injection of steroid in two cases (one was followed by intravitreal dexamethasone implant a few months later). Table 2 shows the cumulative incidence of all complications over 12 months after surgery. Notably, there were no cases of endophthalmitis, IOL disenclavation, subluxation, or dislocation, while IOL tilting or decentration was reported in four eyes (three in group A). The incidence of each type of complication was comparable between the two groups. Univariate and multivariable stepwise linear regression analyses were performed on the final BCDVA, which was predicted most strongly by the BCDVA at 1 month after surgery. The final BCDVA did not show an association with the IOL placement type (anterior vs. retropupillary; Table 4).

## 4. Discussion

Although some previous studies described using iris-claw IOL implantations, very few compared the AC and PC positions [15–17]. The need for a thorough comparison including variables such as ECD and CMT prompted us to perform the present study [18], where we assessed long-term functional and safety outcomes in a large group of eyes that underwent AC or PC iris-claw IOL implantation. We found that both positions afforded significant BCDVA improvement at early (1 month) and long-term (12 months) follow-ups, with final visual and refractive outcomes being similar between the two groups. Concerning safety, CMT, ECD loss, and type and percentage of postoperative complications were comparable between the groups. At the end of follow-up, the CMT was not significantly different from the preoperative value in either group, which was consistent with previous studies [11, 12]. We analyzed ECD loss at 1 and 12 months; loss at the first time point is assumed to mainly reflect intraoperative cellular loss, while ECD loss occurring between 1 month and 12 months is attributable to the presence of the IOL. As expected, the ECD loss rate at 1 month was approximately twice that observed thereafter; however, there was no difference between the two groups in the ECD loss over time. In a previous study comparing AC and PC iris-claw IOLs in combination with PPV, no difference in ECD loss was found at 3 months [17]. Conversely, another study in patients undergoing penetrating keratoplasty, IOL removal, and iris-claw IOL fixation documented a greater ECD loss between 6 months and 12 months after surgery in case of AC IOL placement [16]. The cumulative incidence of all other recorded postoperative complications was similar between the two groups although there was a trend toward a higher proportion of chronic IOP elevation in the AC versus PC IOL position (*p*=0.08). Previous studies evaluating long-term outcomes of iris-claw IOLs reported variable postoperative complication rates [7, 8, 10, 11]. To understand this variability, the surgical procedures performed in conjunction with IOL implantation should be considered, such as complicated cataract surgery or PPV. The relatively high incidence of complication of our groups was comparable to those in a study evaluating iris-claw IOL fixation in conjunction with PPV and lens/IOL removal [17]. Similarly, in our study, the majority of the surgeries (70%) were performed in combination with lens or conventional IOL removal. The elder age of our patients might also have contributed to the increased proportion of complications.

Multivariable analysis of the whole cohort indicated that the BCDVA at 1 month predicted the final BCDVA (i.e., that at the 12-month follow-up). Assuming that variables such as irregular astigmatism, perioperative complications, and preexistent macular or corneal anomalies may influence visual acuity at both 1 and 12 months after surgery, it was expected that good visual acuity at 1 month would persist to 12 months. Notably, the majority of the eyes in our study still had corneal sutures after 1 month. Therefore, we suggest that, for optimal outcomes, astigmatism control should be ensured during application of the corneal suture, irrespective of whether an AC or PC iris-claw IOL is used. The major weakness of our study lays in its retrospective design and the consequent lack of randomization to IOL placement groups or standardization of data collection.

## 5. Conclusion

The present study compared AC and PC iris-claw IOL implantation outcomes over a 12-month follow-up period. Our results showed that the procedures were equally effective and safe for managing cases of aphakia with inadequate capsular support. Future studies should include ultrasound biomicroscopy assessments of the anatomy of AC and PC structures after implantation. Furthermore, in cases of posterior luxation of the lens during cataract surgery, primary iris-claw IOL implantation and a two-step procedure including correction of aphakia at a later and quieter stage should be compared.

## Figures and Tables

**Table 1:** Demographic and baseline characteristics in the two groups.

Patients' characteristics	Group A (*n*=28)	Group B (*n*=32)	*p* value
Age, years	72.7 ± 13.5	73.8 ± 13.4	N.S.
Males, *n* (%)	20 (71%)	21 (66%)	N.S.
Right eyes, *n* (%)	17 (61%)	14 (44%)	N.S.
IOP, mmHg	15.7 ± 5.1	16.2 ± 4.3	N.S.
Preexistent corneal pathology, *n* (%)	3 (11%)	4 (12.5%)	N.S.
Preexistent macular pathology, *n* (%)	2 (7%)	2 (6%)	N.S.
Preexistent retinal pathology, *n* (%)	8 (29%)	11 (34%)	N.S.
Prior closed-globe trauma, *n* (%)	3 (11%)	11 (34%)	0.03
Prior open-globe trauma, *n* (%)	2 (7%)	5 (16%)	N.S.
Prior cataract surgery, *n* (%)	18 (64%)	17 (53%)	N.S.

*Preoperative lens status*			
Subluxated cataract, *n* (%)	5 (18%)	4 (12.5%)	N.S.
Dislocated nucleus, *n* (%)	5 (18%)	11 (34%)	
Subluxated IOL, *n* (%)	6 (21%)	5 (16%)	
Dislocated IOL, *n* (%)	1 (3.5%)	4 (12.5%)	
Opacified IOL, *n* (%)	1 (3.5%)	0 (0%)	
Aphakia, *n* (%)	10 (36%)	8 (25%)	

Continuous variables are presented as means ± standard deviation. *n*: number; IOP: intraocular pressure; IOL: intraocular lens; N.S.: not significant, *p* > 0.05.

**Table 2:** Cumulative incidence of postoperative complications in the two groups over 12 months after surgery.

Postoperative complication	Group A	Group B	*p* value
Cystoid macular edema (%)	33	25	N.S.
Transiently raised IOP (%)	32	22	N.S.
Pseudophakic bullous keratopathy (%)	7	16	N.S.
Epiretinal membrane (%)	7	16	N.S.
Persistent IOP elevation (%)	18	3	N.S. (0.08)
IOL tilting or decentration (%)	11	3	N.S.
Iritis (%)	7	3	N.S.
Retinal detachment (%)	0	3	N.S.
Endophthalmitis (%)	0	0	
IOL disenclavation, subluxation, or dislocation (%)	0	0	

IOP: intraocular pressure; IOL: intraocular lens; N.S.: not significant, *p* > 0.05.

**Table 3:** Outcomes variables at different time points in the two groups.

	Group A (*n*=28)	Group B (*n*=32)	*p* value
Preop. BCDVA (logMAR)	0.66 ± 0.60	0.80 ± 0.66	N.S.
1-month postop. BCDVA (logMAR)	0.35 ± 0.30	0.50 ± 0.50	
1-year postop. BCDVA (logMAR)	0.34 ± 0.45	0.37 ± 0.50	
*p* value (preop. to 1 month, preop. to 1 year, 1 month to 1 year)	0.019, <0.001, N.S.		

*Refractive components*	*n*=28	*n*=32	
1-year postop. Sphere, D	−0.46 ± 0.65	−1.02 ± 1.51	N.S.
1-year postop. Cylinder, D	1.02 ± 1.51	1.08 ± 0.43	N.S.

*CMT*	*n*=21	*n*=27	N.S.
Preop. CMT (*µ*m)	227 ± 64	214 ± 54	
1-month postop. CMT (*µ*m)	229 ± 61	233 ± 99	
1-year postop. CMT (*µ*m)	273 ± 158	229 ± 79	
*p* value (any time interval)	N.S.		

*ECD*	*n*=25	*n*=25	N.S.
Preop. ECD, number of cells (mm^2^)	2043 ± 647	2047 ± 489	
1-month postop. ECD, number of cells (mm^2^)	1721 ± 566	1605 ± 521	
1-year postop. ECD, number of cells (mm^2^)	1512 ± 588	1395 ± 380	
*p* value (preop. to 1-month, preop. to 1-year, 1-month to 1-year)	<0.01, <0.01, N.S.		

Continuous variables are presented as means ± standard deviation. *n*: number; BCDVA: best-corrected distance visual acuity; D: diopter; CMT: central macular thickness; ECD: endothelial cell density; N.S.: not significant, *p* > 0.05.

**Table 4:** Univariate and multivariable regression analysis showing factors influencing final BCDVA.

Covariate	Interval	Univariate analysis		Multivariable analysis	
		*β* coeff	95% CI	*β* coeff	95% CI
Age	1 year increment	0.06	−0.02–0.16	—	
Gender	Female vs. male	0.32	0.07–0.57	0.14	−0.05–0.34
Iris-claw IOL placement	AC vs. PC	0.02	−0.22–0.27	−0.07	−0.26–0.11
Prior trauma	Vs. no prior trauma	0.01	−0.08–1.1	—	
Previous surgery	Vs. no surgery	0.13^*∗*^	0.05–0.20	0.08^*∗*^	0.02–0.14^#^
Preexistent corneal pathology	Vs. no preexistent corneal pathology	0.24	−0.13–0.62	—	
Preexistent macular pathology	Vs. no preexistent macular pathology	0.68^*∗*^	0.22–1.14	0.21	−0.16–0.59
Preexistent retinal pathology	Vs. no preexistent retinal pathology	0.11	−0.15–0.37	—	
Postop. CME	Vs. no postop. CME	0.07	−0.19–0.35	—	
Postop. PBK	Vs. no postop. PBK	0.25	−0.12–0.63	—	
Postop. complication†	Vs. no postop. complications	0.25^*∗*^	0.01–0.49	—	
BCDVA at 1 month	0.1 logMAR increment	0.70^*∗*^	0.48–0.92	0.63^*∗*^	0.42–0.84^#^

^*∗*^ *p* < 0.05; † = excluded from multivariable model due to variance inflation; # = *β*-coefficient based on stepwise regression model for best fit. *R*^2^ = 0.57; IOL: intraocular lens; CME: cystoid macular edema; PBK: pseudophakic bullous keratopathy; BCDVA: best-corrected distance visual acuity; AC: anterior chamber; PC: posterior chamber; CI: confidence interval.

## Data Availability

The data used to support the findings of this study are available from the corresponding author upon request.
